# Machine learning aided jump height estimate democratization through smartphone measures

**DOI:** 10.3389/fspor.2023.1112739

**Published:** 2023-02-09

**Authors:** Guido Mascia, Beatrice De Lazzari, Valentina Camomilla

**Affiliations:** ^1^ Department of Movement, Human and Health Science, University of Rome “Foro Italico”, Rome, Italy; ^2^ Interuniversity Centre of Bioengineering of the Human Neuromusculoskeletal System, University of Rome “Foro Italico”, Rome, Italy

**Keywords:** neural network, accelerometer, gyroscope, strength and conditioning, modal analysis

## Abstract

**Introduction:**

The peak height reached in a countermovement jump is a well established performance parameter. Its estimate is often entrusted to force platforms or body-worn inertial sensors. To date, smartphones may possibly be used as an alternative for estimating jump height, since they natively embed inertial sensors.

**Methods:**

For this purpose, 43 participants performed 4 countermovement jumps (172 in total) on two force platforms (gold standard). While jumping, participants held a smartphone in their hands, whose inertial sensor measures were recorded. After peak height was computed for both instrumentations, twenty-nine features were extracted, related to jump biomechanics and to signal time-frequency characteristics, as potential descriptors of soft tissues or involuntary arm swing artifacts. A training set (129 jumps – 75%) was created by randomly selecting elements from the initial dataset, the remaining ones being assigned to the test set (43 jumps – 25%). On the training set only, a Lasso regularization was applied to reduce the number of features, avoiding possible multicollinearity. A multi-layer perceptron with one hidden layer was trained for estimating the jump height from the reduced feature set. Hyperparameters optimization was performed on the multi-layer perceptron using a grid search approach with 5-fold cross validation. The best model was chosen according to the minimum negative mean absolute error.

**Results:**

The multi-layer perceptron greatly improved the accuracy (4 cm) and precision (4 cm) of the estimates on the test set with respect to the raw smartphone measures estimates (18 and 16 cm, respectively). Permutation feature importance was performed on the trained model in order to establish the influence that each feature had on the outcome. The peak acceleration and the braking phase duration resulted the most influential features in the final model. Despite not being accurate enough, the height computed through raw smartphone measures was still among the most influential features.

**Discussion:**

The study, implementing a smartphone-based method for jump height estimates, paves the way to method release to a broader audience, pursuing a democratization attempt.

## Introduction

The human lower-body neuromuscular ability of generating power, its neuromuscular capacity, readiness, and fatigue can be identified through a vertical jump test (1,2). One of the most commonly used tests for such an analysis is the countermovement jump (CMJ) (1,3). The choice of the CMJ test is supported by its easy familiarization. Furthermore, the CMJ allows the extraction of a plethora of information about the ability of an individual to execute a stretch-shortening cycle (1,3,4). Such a mechanism, occurring at the muscle-tendon level, consists of an eccentric contraction followed by a concentric one of the same muscle group. The elastic enhancement produced throughout the eccentric phase allows to augment the force produced during the concentric one (4). Among the many possible performance parameters biomechanically linked with the jump (5), the most widely analyzed for the CMJ is the reached peak height (1,6).

As a common practice for such an evaluation, force platforms (FPs) and motion capture systems represent the gold standard equipment to estimate the jumper center of mass (CoM) kinematics. The former exploits the ground reaction force (7), whereas the latter directly tracks the CoM displacement (8). Nonetheless, they are generally costly and cumbersome, with limited portability in an outdoor environment, eventually constraining measures to laboratory settings only. In the last few years, inertial measurement units (IMUs) proved to be a valuable alternative to FPs for jump height estimate (9–13). An IMU is composed of two triaxial sensors, namely an accelerometer and a gyroscope, measuring the net force acting on the sensors and their rate of change in angular velocity, respectively (14). The CoM behavior during a jump can be reliably tracked (15) using IMU-measured vertical acceleration by means of an appropriate sensor attachment and position (16), typically a belt worn at pelvis level, and mathematical manipulation (11). The rapid rise and the consequent cost reduction of smartphone devices (SP), which natively embed IMUs, suggests that such an analysis may be accomplished using them. Indeed, SPs are devices within everyone’s reach, which can be thought to be an even more low-cost alternative if compared to commercially available IMUs systems (17). Smartphones would allow democratizing the access to vertical jump tests to every user owning one, especially if performed with no additional equipment (e.g., a belt or pocket holding, etc). Nevertheless, such embedded sensors were not developed for biomechanical analysis, and they do not necessarily present some required specifications, such as high sampling frequency or appropriate full scale range (17).

Smartphone IMUs have already been used to characterize jump activity: to detect it among other activities (18), to find possible correlations between jump mat variables and kinematic features in both CMJ and SJ (19), and to analyze drop jumps (20,21). Estimating jump height using SP-embedded IMU direct measures has not been attempted, to the best of our knowledge, aside from preliminary investigations forerunner of the current one (22,23). SP-focused studies were all based on video camera-based approaches (24,25). Among the alternatives available on the market, the most commonly used SP application is MyJump, whose reliability has been tested in several studies (26–32). Nonetheless, it uses a method relying on specific assumptions about the symmetry of the task execution, and it computes jump height using flight time, entailing the identification of take-off and landing instants of time. Developing jump height estimates through an SP-embedded IMUs, besides coping with low performance embedded sensors, requires framing the role of three factors and possibly compensate for their negative consequences: signal-processing procedures, sensor position, and presence of signal artifacts due to soft tissues (17,33).

Vertical jump height can be computed using three main *signal-processing procedures*: take-off velocity method (TOV), flight time method (FT), and direct tracking of CoM trajectory using motion capture systems (34). When using IMU data, only TOV and FT can be used. Evidence shows that TOV outperforms FT in terms of both biomechanical accuracy (35–38) and reliability (1,7,36,37). Indeed, FT involves the identification of two instants of time (take-off and landing), whether TOV requires only one (take-off). Moreover, FT is dependent on the hypothesis that the peak height is reached at exactly half the flight-time: this is not the case if the jumper naturally folds their legs, thus extending the flight-time, leading to a height overestimation (35–38). In the context of a self-evaluation with no external supervision, this issue could be critical. As regards experimental errors, estimating velocity from IMUs through numerical integration is usually affected by sensor errors. However, since CMJ entails a short integration interval (≃1.5 s from the movement onset to the take-off instant - (5)), this error can be considered negligible (12,39,40). Hypothesizing errors due to velocity estimation comparable or lower than those due to time events identification, preference is given to the TOV method, rather than the TF, since it: (i) involves the identification of a single time instant; (ii) is not affected by the jumper postural configuration.

**Figure 1:**
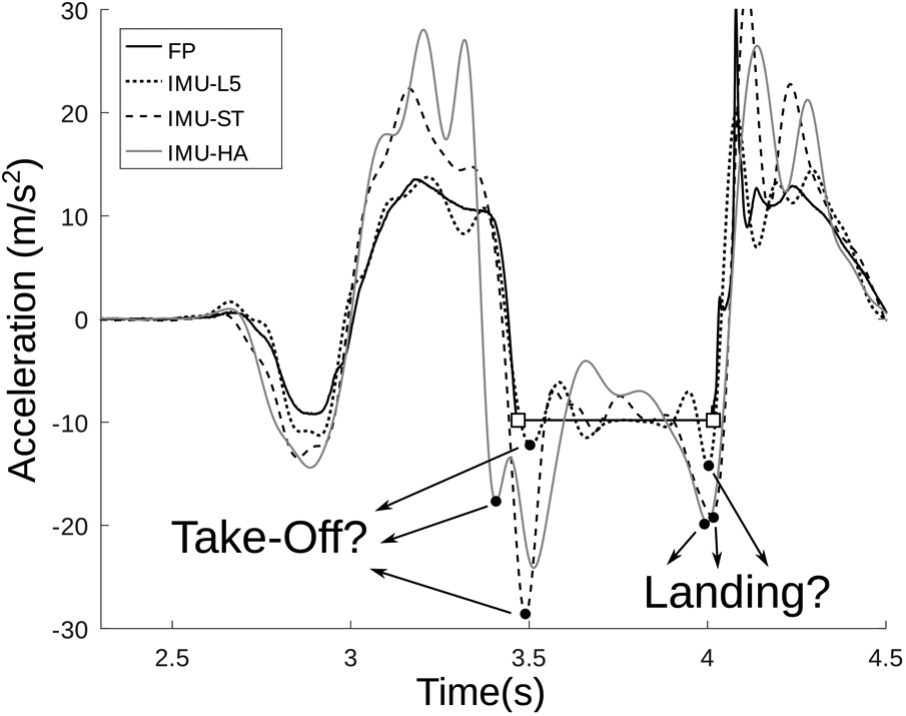
Example of vertical acceleration during CMJ, measured at different positions and instrumentations. Legend: Force Platform gold standard (continuous line, FP); IMU at L3-L5 vertebrae (dotted line, IMU-L5); IMU attached close to the sternum (dashed line, IMU-ST); IMU placed within the hands (gray continuous line, IMU-HA) resembling the configuration used in the study; the square markers identify the actual take-off and landing instants on the FP signal. IMU signals were low-pass filtered (Butterworth, 6th order, $f_{c}=10$ Hz). It is possible to appreciate the wide variability of the key time instants (take-off and landing) depending on the sensor position. The depicted data were acquired through Vicon Nexus software using Vicon Blue-Trident IMUs (Vicon, Oxford, United Kingdom; sampling frequency: 1,000 sample/s; full scale range: accelerometer = ±16 g, gyroscope = ±2,000 deg/s).

The impact on jump height estimation accuracy of *sensor position* depends on several factors: first, measuring the acceleration at a single point close to the ideal CoM location (L3-L5 vertebrae) neglects the CoM displacement associated with the relative movements of body masses (41) (Figure 1). Second, if the sensing element is far from this point, e.g. attached to the sternum, the acceleration shape recorded during a jump is further different (Figure 1) (15). Nevertheless, the cumbersome nature of SPs makes their placement in proximity to the ideal human CoM position unsuitable. Holding the SP within the hands constitutes an ecological alternative, but may further increase this discrepancy. Achieving comparable results to the ones obtained through belt-worn IMUs (mean difference ≃5 cm - (10)) calls for developing ad hoc methods that compensate for the above-mentioned discrepancies.

*Soft tissue artifacts*, considered the most detrimental factor in human movement analysis (33), are of no second importance also for IMU measures (42,43). This is particularly true for high intensity movements, which presumably enhance wobbling due to the inertial whiplash of the soft tissues and of the attached sensor, depending on the sensor fixing (14,16). Although wobbling has been claimed as a secondary artifact cause, being accounted for a relatively small part of the total mean power (on average, 13% for 3 m/s running (44)), it can still be assumed to have an important role in disrupting the consistent estimate of both take-off and landing time instants (Figure 1), further reinforcing the choice of using TOV as preferential approach. Moreover, the disruptive effects brought to the signal waveform are potentially overlapped to the motor task in the frequency domain (44). As a promising alternative to characterize such effects, the use of modal analysis in the form of variational mode decomposition (VMD) could be beneficial. VMD is a numerical method able at discriminating a selected number of intrinsic mode functions, each centered around a specific central frequency (45). Such a technique could help in highlighting the oscillatory time-frequency traits of the analyzed signals, discriminating contributions attributable to different wobbling elements.

This study aims to estimate jump height using SP-embedded IMU measures. To cope with given uncertainties related to low performance sensors, sensor position, and soft tissues artifacts, jump height is predicted on the basis of selected information extracted from these measures. Features must be chosen as appropriate for the context they are describing: those biomechanically linked with the jump (5) would be reasonable candidates for obtaining a valid estimate; but it is also essential to embed features that can be predictive, and thus possibly compensate for, the wobbling oscillations mentioned above. This is in line with recent trends having data science emerging as a discipline capable of supporting findings related to sports-related issues through the use of automated methods (46–48).

A feature set was selected to estimate jump height through supervised learning. Gold standard height obtained via FP data and TOV method was used as reference outcome. Possible multicollinearity between the selected features was eventually tackled by using Lasso regularization. Considering only the reduced feature set, a machine learning approach was alternatively investigated training a multilayer perceptron neural network (MLP) through hyperparameters tuning performed via grid search. The influence that each feature exerted over the outcome was also evaluated using the permutation feature importance technique (PFI) (49).

## Materials and methods

### Experimental setup

**Figure 2:**
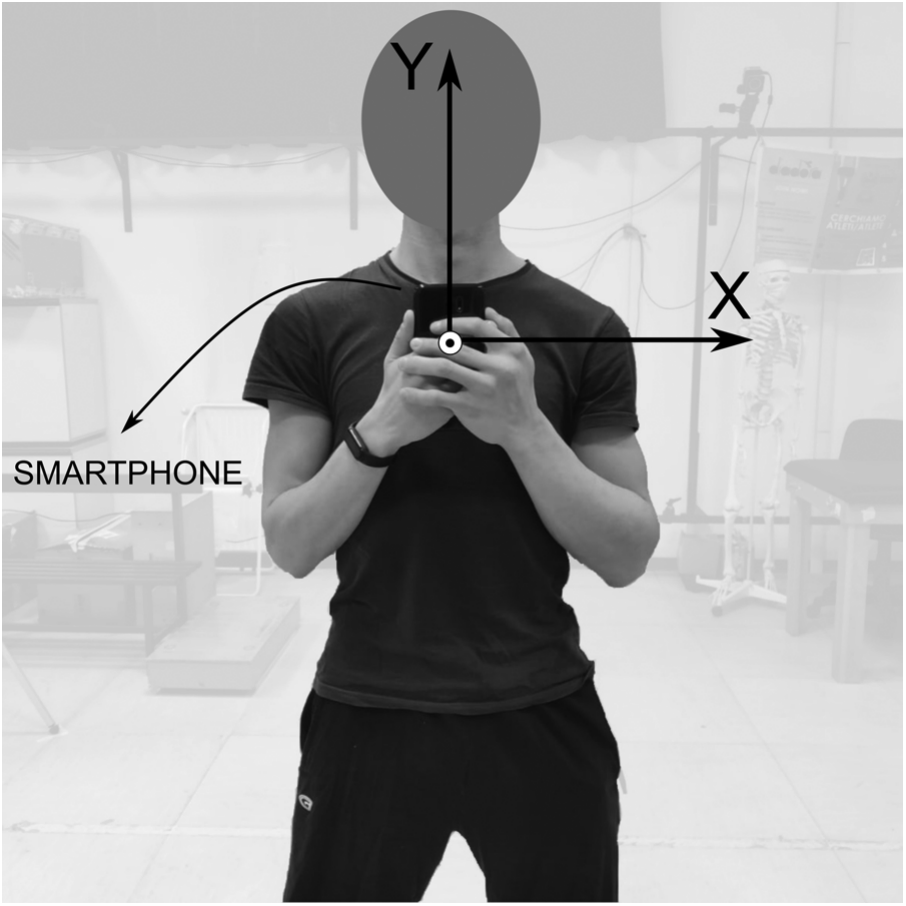
The experimental setup utilized for acquiring data from the SP device using the app Phyphox (remotely controlled via PC).

Forty-three healthy sports science students were recruited (27M, 16F; mean±SD: age=25.9±3.7 years; stature=171±10 cm; mass=67.5±10.9 kg). All participants signed an informed consent prior to the experimental session. Only physically active individuals were included, whereas individuals which underwent either lower limb surgery or injury in the six months prior to the experimental session were excluded from the study. The study was approved by the Internal Review Board of the University of Rome “Foro Italico” (No. CAR 94/2021). Participants held an SP in their hands (Figure 2) (Xiaomi Redmi 9T, Beijing Xiaomi Technology Company Limited, Beijing, China; sampling frequency = 128 samples/s; full scale range: accelerometer=±8 g; gyroscope=±360 deg/s). All SP-IMU data were collected using the app Phyphox (50), which was controlled remotely through the laboratory PC. Calibration tests were performed before each experimental session. These operations are essential to re-align the vertical acceleration signal with the world reference frame through sensor fusion algorithm, detailed in the “Data processing” section. Afterwards, each participant was instructed on how to properly perform one round of the experimental task according to the recommendations in (1). Then, they performed 4 CMJs onto two FPs (AMTI, Watertown, Massachusetts, USA; sampling frequency = 1,000 samples/s; size=40×40 cm), one under each foot. Jumps were executed with the elbows at waist height, to limit arm swing inertial effects and to comfortably hold the SP with the hands (Figure 2). Each CMJ was visually inspected to verify if compliant with the prescribed recommendations and, if not, it was excluded. The task was executed over two FPs since: (i) a single one was too small (40×40 cm) for consistently recording a complete CMJ (static, loading, and landing); (ii) with two FPs the jumper attention was focused on task execution, rather than on landing on the FP. Each jump consisted of: (i) a static phase of a few seconds with the participant being in orthostatic position; (ii) a vocal command signaling the jump initiation; (iii) a second static phase as in (i).

### Data processing

The SP-IMU offset and cross-axis sensitivity were computed and corrected according to Bergamini et al. (51) before each experimental session. Vertical acceleration measures were aligned to the world reference to allow for a consistent gravity removal (11). Only the vertical component of the acceleration, $a^{\mathrm{SP}}$, was considered for further computations.

In particular, gyroscope static bias was extrapolated from a 60 s static trial with the SP still on a flat surface, hence removed from each successive jump measure. For what concerns the accelerometer, three ad hoc 60 seconds acquisitions were performed; each consisted in aligning one of the three accelerometer axes with the gravity vector direction (51).

A comparable vertical acceleration measure, $a^{\mathrm{FP}}$, was obtained from FP data. First, participant mass (m) was computed averaging the first second of the first static phase and dividing it by g (g=9.81 m/s${}^{2}$). Consequently, the whole ground reaction force was divided by m, hence g was subtracted to obtain $a^{\mathrm{FP}}$.

Peak jump height was computed through the TOV method for both instruments ($h^{\mathrm{SP}}$ and $h^{\mathrm{FP}}$, respectively). For both data sets, vertical velocity, v, was computed through numerical integration of the corresponding acceleration from the CMJ onset ($t_{0}$) to the take-off ($t_{\mathrm{TO}}$). More specifically: $t_{0}$ was computed as the time sample occurring 30 ms prior the first one deviating by 8 times the static phase standard deviation, following a similar approach as in Owen et al. (52); $t_{\mathrm{TO}}$ was chosen to be the first time sample such that a≤−g. All data were processed and analyzed using GNU Octave (53).

### Feature selection

**Figure 3:**
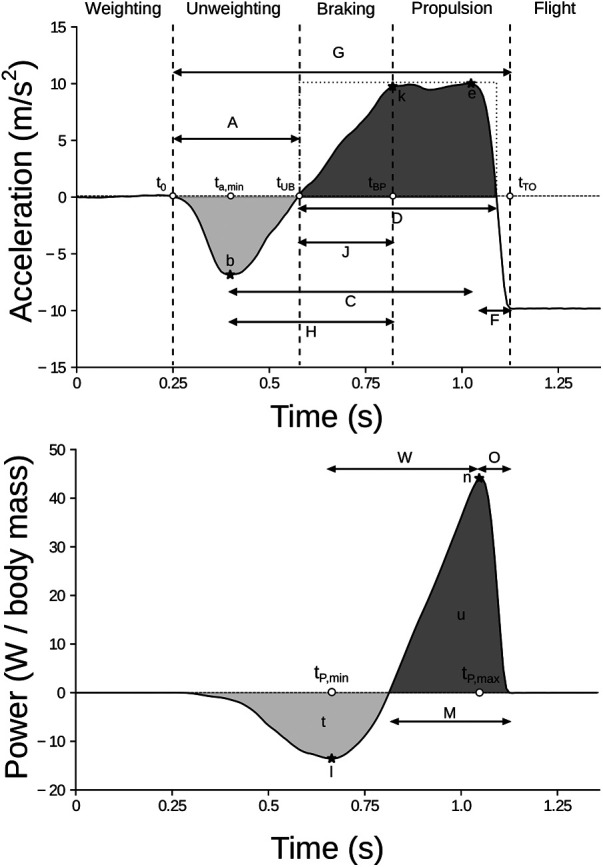
Visual depiction of the selected features, for a representative jump. Acceleration-related features are shown in the top panel, while power-related ones in the bottom panel. The vertical dotted lines in the top panel represent jump-phases transitions (1): the weighting phase lasts from the beginning to $t_{0}$ (jump onset); the unweighting phase lasts from $t_{0}$ to $t_{\mathrm{UB}}$; the braking phase lasts from $t_{\mathrm{UB}}$ to $t_{\mathrm{BP}}$; the propulsion phase lasts from $t_{\mathrm{BP}}$ to $t_{\mathrm{TO}}$. Notice that $t_{\mathrm{UB}}$ and $t_{v,\min}$ coincide. For the sake of clarity, only the former was depicted. Feature i cannot be represented as its numerical value was derived from further computations. The meaning of each feature is detailed in Table 1.

**Figure 4:**
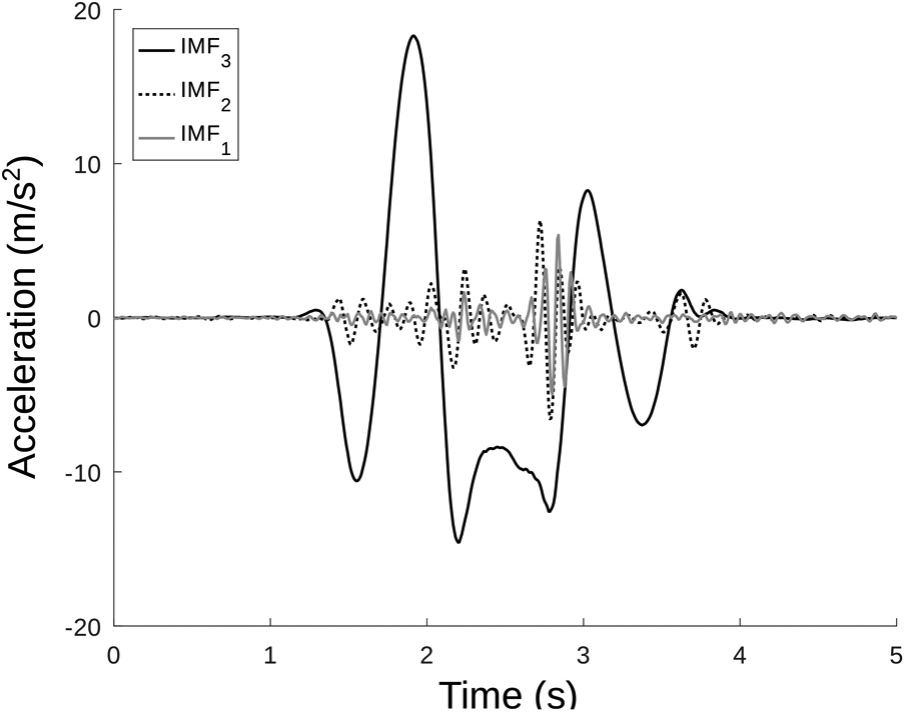
Intrinsic mode functions (IMFs) resulting from the application of the VMD algorithm on a representative CMJ. The black, dashed line represents the high frequency IMF${}_{1}$; the gray continuous line represents the middle frequency IMF${}_{2}$; the black continuous line represents the low frequency IMF${}_{3}$. In this specific case: $f_{1}=6.06$ Hz; $f_{2}=3.49$ Hz; $f_{3}=0.55$ Hz.

A total of M=26 features were extracted, as detailed in section “Model creation and evaluation”. All the listed features are depicted in Figures 3, 4 and detailed in Table 1. All of them were extracted from $a^{\mathrm{SP}}$, including the raw estimate of the height, $h^{\mathrm{SP}}$. Nineteen jump-related variables (features from A to s) were inspired by Dowling and Vamos (5); three of them (u, W, and z), enlarging the description of power related variables were presented in Mascia and Camomilla (22); finally, the last three features are the central frequencies obtained processing $a^{\mathrm{SP}}$ via VMD (45), subdividing the signal into three intrinsic mode functions, each having a frequency spectrum centered around each of them. In particular, the high- and mid-central frequencies ($f_{1}$ and $f_{2}$) were assumed to be associated with the inertial effects due to wobbling masses, whereas the low-central frequency ($f_{3}$) was thought to be associated with the jump itself (Figure 4).

**Table 1:** Detailed explanation of each of the analyzed features.

ID	Feature	Measure unit	Description
$h^{\mathrm{SP}}$	Jump height	m	Height computed via TOV from $a^{\mathrm{SP}}$
A	Unweighting phase duration	s	$\left[t_{0},t_{\mathrm{UB}}\right]$
b	Minimum acceleration	m/s${}^{2}$	$a(t_{a,\min})$
C	Time from minimum to maximum acceleration	s	$\left[t_{a,\min},t_{a,\max}\right]$
D	Main positive impulse time	s	Time duration of positive acceleration from $t_{\mathrm{UB}}$ to the last positive sample prior $t_{\mathrm{TO}}$
e	Maximum acceleration	m/s${}^{2}$	$a(t_{a,\max})$
F	Time from acceleration positive peak to take-off	s	$\left[t_{a,\max},t_{\mathrm{TO}}\right]$
G	Ground contact duration	s	$\left[t_{0},t_{\mathrm{TO}}\right]$
H	Time from minimum acceleration to the end of breaking phase	s	$\left[t_{a,\min},t_{\mathrm{BP}}\right]$
i	Maximum positive slope of acceleration	m/s${}^{3}$	max(da(t)/dt), $t\in [t_{a,\min},t_{a,\max}]$
k	Acceleration at the end of the braking phase	m/s${}^{2}$	$a(t_{\mathrm{BP}})$
J	Time from negative peak velocity to the end of breaking phase	s	$\left[t_{v,\min},t_{\mathrm{BP}}\right]$
l	Negative peak power	W/kg	$P(t_{P,\min})$
M	Positive power duration	s	Self-explanatory
n	Positive peak power	W/kg	$P(t_{P,\max})$
O	Time distance between positive peak power and take-off	s	$\left[t_{P,\max},t_{\mathrm{TO}}\right]$
p	Mean slope between acceleration peaks	au	p=(e−b)/C
q	Shape factor	au	Ratio between the area under the curve from $t_{\mathrm{UB}}$ to the last positive sample prior $t_{\mathrm{TO}}$ (lasting D) and the one of a rectangle of sides D and e
r	Impulse ratio	au	r=b/e
s	Minimum negative velocity	m/s	$v(t_{v,\min})$
u	Mean concentric power	W/kg	Average value of P(t), $t\in [t_{\mathrm{BP}},t_{\mathrm{TO}}]$
W	Power peaks delta time	s	$\left[t_{P,\min},t_{P,\max}\right]$
z	Mean eccentric power	W/kg	Average value of P(t), $t\in [t_{0},t_{\mathrm{BP}}]$
$f_{1}$	High central frequency	Hz	Highest VMD central frequency, associated with wobbling tissues and noise
$f_{2}$	Middle central frequency	Hz	Middle VMD central frequency, associated with wobbling tissues
$f_{3}$	Low central frequency	Hz	Lowest VMD central frequency, associated with the jump proper

Capital letters are for timings, small letters for the other parameters. Legend: au = arbitrary units; $t_{0}$,= jump onset time; $t_{\mathrm{UB}}$, unbraking-braking phase transition time; $t_{\mathrm{BP}}$, braking-propulsion phase transition time; $t_{\mathrm{TO}}$, take-off time; $t_{a,\min}$, minimum acceleration time; $t_{a,\max}$, maximum acceleration time; $t_{v,\min}$, minimum velocity time; $t_{P,\min}$,= minimum power time; $t_{P,\max}$, maximum power time.

### Model creation and evaluation

The final dataset was composed by 172 jumps, each associated with the corresponding 26 features computed from $a^{\mathrm{SP}}$, and the peak height computed from $a^{\mathrm{FP}}$, $h^{\mathrm{FP}}$, considered as the dependent variable. Once data was arranged for all the jumps, the dataset was separated in two. More specifically, 75% of the jumps (129 examples) was used for creating the training set, whereas the remaining 25% (43 examples) was used as test set (54,55). The elements belonging to each of the two subsets were randomly selected.

Before training, each feature of the training set was normalized via z-score (55). The mean values and the standard deviations of each feature distribution were stored, so that they could be successively used for normalizing the test set features, accordingly.

In order to avoid possible multicollinearity between features, a feature reduction approach was performed on the training set using Lasso regularization (56). The regularization strength was set by selecting a value of α=0.1. The features that were excluded from such a shrinkage were not used for developing the machine learning algorithm.

A multilayer perceptron neural network (MLP) with one hidden layer was then used as machine learning architecture. A grid search with 5-fold cross validation was used for tuning the model hyperparameters (55,57). In particular, three hyperparameters were tuned: the activation function, the solver algorithm, and the number of neurons composing the hidden layer. The complete list of all the candidate hyperparameters for the grid search are presented in Table 2. The best and final model was chosen as the one with the combination of hyperparameters ensuring the best negative mean absolute error (58).

**Table 2:** List of possible choices for each hyperparameter during the grid search.

Hyperparameter	Choices
Activation function	Identity, logistic, tanh, ReLU
Solver algorithm	lbfgs, sgd, adam
No. neurons in hidden layer	1–16

Legend: tanh, hyperbolic tangent; ReLU, rectified linear unit; lbfgs, limited-memory Broyden-Fletcher-Goldfarb-Shanno algorithm; sgd, stochastic gradient descent.

The height computed through the MLP was denoted as $h^{\mathrm{MLP}}$, and compared also to the height directly estimated using the SP, $h^{\mathrm{SP}}$.

To obtain a quantitative description of the influence that each feature had on the outcome, a permutation feature importance analysis (PFI) was performed on the trained model (49). Lets consider the model $\hat{\;f}$, the feature matrix X with j as feature index, the target $h^{\mathrm{FP}}$, and the error $L[h^{\mathrm{FP}},\hat{\;f}(X)]$. In this study, the mean squared error (MSE) was selected as model error, since it enhances the prominence of feature importances being quadratic. Each permutation was performed on the training set only, meaning that each feature importance ${\mathrm{FI}}_{\;j}$ here presented belong to that set. Moreover, PFI was accomplished by splitting such a set in half, according to the recommendations proposed in Fisher et al. (59). The PFI algorithm follows:

1. Estimate the original model error: $e_{0}=L[h^{\mathrm{FP}},\hat{\;f}(X)]$.
2. ∀ feature j∈{1,…,M}:
  2.1 Generate the feature matrix with the permuted feature $X_{\mathrm{perm}}^{\;j}$.
  2.2 Estimate the error for the feature matrix with the permuted jth feature: $e_{j}=L[h^{\mathrm{FP}},\hat{\;f}(X_{\mathrm{perm}}^{j})]$.
  2.3 Compute the feature importance as: $FI_{j}=e_{j}/e_{0}$.

### Statistical analysis

The performances of the criteria used for estimating $h^{\mathrm{SP}}$ and $h^{\mathrm{MLP}}$ were evaluated exploiting three metrics: (i) accuracy, considered as the root mean squared distance (RMSD) between the estimates and the true values; (ii) bias, computed as the average difference between the estimates and the true values; (iii) precision, representing the standard deviation of the differences, i.e., the dispersion of the error. Metrics (ii) and (iii) were directly computed from Bland-Altman plot analysis (60). Furthermore, the mean absolute error (MAE) was computed as an overall performance metrics for both the models.

Kendall’s Tau test (61) was used for exploring possible heteroscedasticity of $h^{\mathrm{SP}}$ and $h^{\mathrm{MLP}}$ estimates (62,63). More specifically, the test was performed comparing the distribution of the averages versus the absolute differences of the gold standard and predicted values, as in Brehm et al. (63). If τ<0.1, data were considered homoscedastic; conversely, if τ≥0.1, data were considered heteroscedastic.

Paired sample t-test was performed on the test set comparing the $h^{\mathrm{FP}}$ distribution with the $h^{\mathrm{SP}}$ and $h^{\mathrm{MLP}}$ ones, respectively. Finally, a linear regression analysis between $h^{\mathrm{FP}}$ with $h^{\mathrm{SP}}$ and $h^{\mathrm{MLP}}$, respectively, was performed, so that a calibration between the systems could also be provided.

All the procedures regarding model creation and its performance evaluation have been accomplished through the *scikit-learn* environment (58).

## Results

A total of 172 jumps were analyzed. Jumps height measured through the gold standard FP ranged from 10 cm up to 41 cm (25.6±7.4 cm).

From the initial set of 26 features, only 17 were included in the final model after Lasso regularization. In particular, the features excluded were the following: A,C,D,k,m,n,o,p, and $f_{2}$, which meaning is explained in Table 1.

The grid search procedure analyzed a total number of 192 (4 activation functions × 3 solver algorithms × 16 neurons in the hidden layer) possible hyperparameter combinations. The best one (i.e., the one presenting the best negative mean absolute error among the 5 folds) was found to be the one having a logistic activation function with an hidden layer composed of 11 neurons, and its solver being the stochastic gradient descent algorithm. Among the 5 folds, the selected combination of hyperparameters presented an average negative mean absolute error (±SD)=−0.8±0.0 cm, with an average $R^{2}=0.978$. The results obtained for each of the 5 folds are detailed in Table 3.

**Table 3:** Scores of the grid search with cross validation for the best model. Notice that such results are related to the training set only.

	Negative MAE (cm)	$R^{2}$
Fold 1	−0.8	0.975
Fold 2	−0.7	0.981
Fold 3	−0.7	0.982
Fold 4	−0.8	0.975
Fold 5	−0.8	0.977
Mean	−0.8	0.978

Accuracy, computed as RMSD between $h^{\mathrm{FP}}$ and the estimates, improved of 4.5 times using the MLP model instead of SP-derived estimates. Similarly, using the MLP rather than the SP improved of 4 times the precision (SD of the differences between measure and estimate) and reduced of 4 times the MAE. The performance analysis for the analyzed models is detailed in Table 4. Moreover, Kendall’s tau analysis performed on the average versus the absolute difference showed that $h^{\mathrm{SP}}$ distributions presented heteroscedasticity (τ=0.38). On the contrary, $h^{\mathrm{MLP}}$ distribution did show homoscedasticity (τ=−0.02).

**Table 4:** Results of the performance analysis for the analyzed models.

	Accuracy (cm)	Bias (cm)	Precision (cm)	MAE ± SD (cm)	τ
SP	18.0	−7.0	16.0	12.0±13.0	0.38
MLP	4.0	−0.0	4.0	3.0±3.0	−0.02

The accuracy is computed as the RMSD of $h^{\mathrm{FP}}$ and the estimates of each model; the bias is computed as the average difference of $h^{\mathrm{FP}}$ and the estimates of each model; the precision is computed as the standard deviation of the differences between $h^{\mathrm{FP}}$ and the estimates of each model; MAE is the mean absolute error (± standard deviation); τ is the Kendall’s tau coefficient of the averages versus the absolute differences for each model.

Paired sample t-test analysis showed significant differences between $h^{\mathrm{FP}}$ and $h^{\mathrm{SP}}$, whereas no significant difference was found between $h^{\mathrm{FP}}$ and $h^{\mathrm{MLP}}$. The results of such an analysis are detailed in Table 5. Finally, the results of the linear regression analysis for calibrating the two systems are provided in Table 6.

**Table 5:** Results of the paired samples t-test analysis performed comparing $h^{\mathrm{FP}}$ with $h^{\mathrm{SP}}$ and $h^{\mathrm{MLP}}$, respectively.

	t value	p value	Degrees of freedom	95% CI (cm)
SP	−2.770	0.008${}^{*}$	42	[−12.2,−1.8]
MLP	0.711	0.481	42	[−1.0,2.0]

Statistically significant differences are indicated with the superscript (${}^{*}$).

**Table 6:** Linear regression analysis performed between $h^{\mathrm{FP}}$ with $h^{\mathrm{SP}}$ and $h^{\mathrm{MLP}}$, respectively.

	Intercept [95% CI] (cm) t value	$h^{\mathrm{SP}}$ [95% CI] (cm) t value	$h^{\mathrm{MLP}}$ [95% CI] (cm) t value	SEE (cm)	r
SP	17.668${}^{***}$ [13.944 21.391] 9.582	0.226${}^{***}$ [0.127 0.326] 4.594	—	6.257	0.583
MLP	5.681${}^{*}$ [0.873 10.488] 2.386	—	0.789${}^{***}$ [0.600 0.977] 8.460	4.647	0.797

Legend: intercept, $h^{\mathrm{SP}}$, and $h^{\mathrm{MLP}}$ are the coefficient of the regression analysis with 95% confidence intervals and t value; SEE is the standard error of the estimates, computed as the standard deviation of the regression residuals; r is the correlation coefficient. Significance level: (${}^{*}$) p<0.05; (${}^{***}$) p=0.

**Figure 5:**
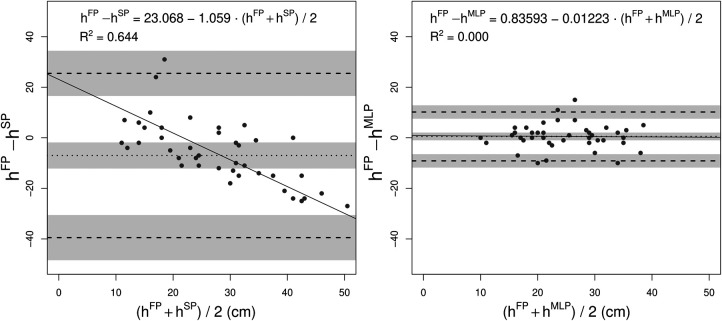
Bland-Altman analysis for the height estimates obtained directly from the SP with the TOV method (left) and through the MLP (right). The continuous line represents the bias (b) computed as the average difference between the gold standard and the estimate; the dot-dashed lines represent the lower and upper confidence intervals (UB and LB, respectively), computed as 1.96 standard deviations of the difference distribution away from the bias, in both directions. The gray shaded areas indicate the confidence interval for b, UB, and LB, respectively. The continuous line is the regression line of the average versus the differences, which equation is indicated in the above portion of the graph. The $R^{2}$ value refers to the latter equation.

Bland-Altman plots of both SP- and MLP-heights estimated for the test set are presented in Figure 5, whereas the corresponding numerical values are reported in Table 7.

**Table 7:** Bland-Altman analysis results for the two compared methods.

	Standardized bias	Bias [95% CI] (cm)	LB [95% CI] (cm)	UB [95% CI] (cm)
SP	−0.3	−7.0 [−12.2,−1.8]	−39.5 [−30.5,−48.4]	25.5 [34.4, 16.5]
MLP	0.0	0.5 [−1.0,2.0]	−9.1 [−11.8,−6.5]	10.2 [7.5, 12.8]

All the values and the related confidence intervals were computed according to what proposed in Giavarina (64). The corresponding graphical depiction of the values is presented in Figure 5.

**Figure 6:**
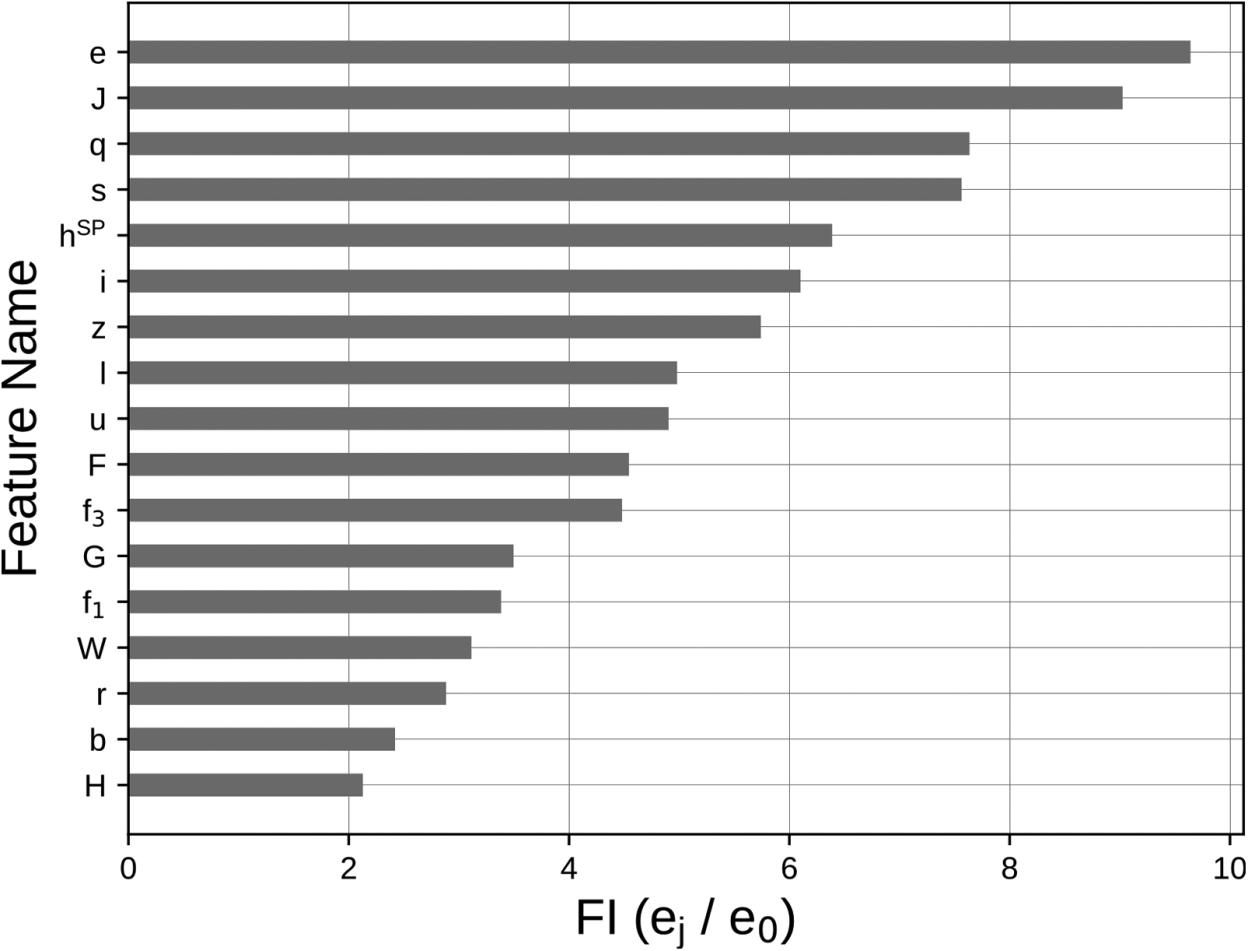
Barchart representing the outcome of the PFI analysis performed on all the variables selected by the Lasso regularization. Variables meaning is detailed in Table 1.

Permutation feature importance analysis highlighted the influence of each variable on the outcome. The five most influential features were e (maximum acceleration, FI=9.64), J (time from negative peak velocity to the end of the braking phase, FI=9.02), q (shape factor, FI=7.63), s (minimum negative velocity, FI=7.56), and $h^{\mathrm{SP}}$ (height computed from SP via TOV, FI=6.39). A bar chart showing all outcomes of the PFI analysis for each variable is presented in Figure 6.

## Discussion

In this study, a step towards jump height democratization using a smartphone through machine learning was proposed. While the SP alone entails unacceptable errors, the neural network model proposed here in the form of an MLP, reduced the error to produce outcomes that could be of use in an in-field setting.

The obtained results are promising considering that the low-cost of the IMU embedded into the smartphone allowed a feature extraction capable of predicting height estimates under the proposed assumptions. Indeed, the errors obtained are comparable to those typically obtained comparing commercially available IMU systems with FPs (mean difference ≃5 cm), as revised in Clemente et al. (10). For what concerns the accuracy, the MLP improved it bringing the discrepancy from 18 cm down to 4 cm. This improvement, as expressed in percentage of the true value, equivals obtaining 15.6% errors. The improvement brought by the MLP similarly improved precision from an initial value of 14 cm down to 4 cm, in line with what obtained in literature (10), acceptable depending on the subsequent application. Moreover, it is possible to appreciate that the MLP provided virtually no systematic bias. On the contrary, the average versus difference plot of $h^{\mathrm{SP}}$ (Figure 5) indicates that, on average, the use of TOV method leds to underestimated jump height values. Finally, an heteroscedastic trend is clearly visible in the error of SP-computed jump heights (Figure 5), as confirmed by Kendall’s Tau analysis (τ=0.38). On the contrary, there is no trend for MLP-estimates (Figure 5, τ=−0.02). Estimates homoscedasticity corroborate model generalizability outside the training dataset.

Feature reduction, performed using Lasso regularization to avoid possible multicollinearity between the initial set of features, selected 17 features as model input. Permutation feature importance analysis proved this reduced feature set as influential of the outcome and ranked it in terms of influence on the model output (Figure 6). All the selected features, once the dataset was permuted (59), increased the MSE from a minimum of 2- to a maximum of about 9-times if compared with $e_{0}$ (see section “Model creation and evaluation”). Indeed, all the features exhibited a FI>1, possibly indicating that Lasso regularization included only those features that could be predictive of jump height.

Among the five most important features, four of them (J,e,q, and s) belonged to two phases of the jump (braking and propulsion phases), with the fifth most important being the height computed through the TOV method from smartphone measures ($h^{\mathrm{SP}}$).

Maximum acceleration (e, typically occuring in the propulsion phase of the CMJ), and the time from negative peak velocity to the end of the braking phase (i.e. the duration of the braking phase – J) were much more influential than all the others, increasing the MSE of about 9 times, when permuted. This could be seen as a mutual relationship, even though not collinear, between two distinct features of the CMJ. Indeed, the correlation between e and J in the training set was r(e,J)=−0.52, meaning that the shorter the braking phase, the bigger the magnitude of the recorded maximum acceleration. Moreover, the minimum negative velocity value (s) importance resulted prominent as well, having an impact of about 7-times on the MSE of the trained model. The impact of these three features together could be possibly linked to the biomechanical mechanism required for achieving the best possible jump height, that is, the stretch-shortening cycle (1,3,4).

The shape factor (q) after PFI influenced the MSE increasing it of about 7 times. This is in line with the claimed potential of this feature to relate to an optimal force generation for achieving the maximum performance (5).

Although the height computed through smartphone alone (feature $h^{\mathrm{SP}}$) is not sufficient for obtaining accurate estimates, it is still one of the most influential features as a first guess value to stay close to (FI=6.39). Even though less influential than the above-mentioned features (FI=4.48), the high central frequency ($f_{3}$) testifies the role of wobbling into disrupting jump height estimates. This is in line with similar results obtained using vertical acceleration measured from a belt-worn IMU at pelvic level: for this signal, the high central frequency was included in a model predictive of jump power, possibly compensating for the effects of soft tissue wobbling (22). This is also promising in the perspective of using VMD to identify the contribution of arm swing into vertical jump height, when voluntarily included in the jump action.

Such results must be analyzed in a wider framework. First, the sampling frequency, considered as a key player in jump height estimates quality, was lower (128 samples/s) than the 1,000 samples/s suggested as essential for a proper jump analysis (52). Nonetheless, testing the worst-case scenario was motivated by the democratization mission of this study, as results would then apply to any SP device available on the market, irrespective of its sensor sampling frequency. Second, holding the sensor in the hands was thought to be the most practical while repeatable experimental setup, with little perturbation to each jumper technique; however, it entails an undesired upper limb oscillatory movement. Despite the central frequency associated with this effect partially compensates for it, it can be hypothesized that placing the SP closer to the trunk and/or within an ad hoc harness could further enhance the prediction. Third, trial height ranged from a minimum of 10 cm up to a maximum of 41 cm, since none of the participants was an elìte athlete. Theoretically, the model output can be considered valid only within this range.

Among the strengths of this study, the model was devised using as gold standard reference jump heights that: (i) were computed from laboratory FP signals; (ii) were estimated through the TOV method, considered as the most reliable (36). Moreover, the approach presented here involves the use of features which are biomechanically related to the nature of the jump itself. Most importantly, they can be easily retrieved directly from the acceleration trace during the jump with simple mathematical manipulation.

The method could be embedded in the creation of a SP application, exploiting the current results to give runtime insights about jump height. The possibility to access the code and the methodology used (github.com/Maskul93/height-democratization) can also leverage actions to enlarge the current database and improve the estimates.

## Data Availability

The raw data supporting the conclusions of this article will be made available by the authors, without undue reservation.
